# Evaluation of Histopathological Risk Model in a Cohort of Oral Squamous Cell Carcinoma Patients Treated with Accompanying Neck Dissection

**DOI:** 10.1007/s12105-021-01326-4

**Published:** 2021-04-22

**Authors:** N. Rahman, B. Conn

**Affiliations:** 1 Edinburgh Dental Institute, Lauriston Building, Lauriston Place, Edinburgh, EH3 9HA UK; 2 Royal Infirmary of Edinburgh, 51 Little France Crescent, Old Dalkeith Road, Edinburgh, EH16 4SA UK

**Keywords:** Oral squamous cell carcinoma, Nodal metastasis, Pattern of invasion, Lymphocytic host response, Perineural invasion

## Abstract

To investigate the applicability of the validated histological risk model in a cohort of oral cavity squamous cell carcinoma patients treated concurrently with neck dissections. Primary tumours from 85 patients with primary excision of T1 and T2 Oral Squamous Cell Carcinomas (TNM 7th edition) including neck dissection were scored by three pathologists in consensus according to the validated risk model. The risk score data, along with traditional dataset values, were analysed to determine possible association with nodal metastasis and extracapsular spread. Seventy-two patients (54%) were classified with low or intermediate risk and 62 (46%) patients were ‘high risk’. A chi squared test showed that cases with nodal metastasis were highly statistically significant with the overall risk model score (*X*^2^ = 22.62 *p* = 0.0001). None of the neck dissections from tumours with low risk score showed evidence of metastasis (NPV = 100%) suggesting the risk score may also be a useful tool for predicting an absence of metastasis. Risk assessment of low-stage oral squamous cell carcinoma primary tumours may be predictive of the presence or absence of metastasis at presentation. Knowledge of the risk score and its constituent parts may inform treatment decisions at multidisciplinary meetings. Low risk squamous cell carcinoma may be a rare variant with low metastatic potential and excellent long-term survival.

## Introduction

The validated histopathological risk model introduced by Brandwein-Gensler and colleagues in 2005 has been shown to have significant prognostic value for low stage OSCC patients. The model places weighted point values on three key subjective histological parameters: worst pattern of invasion (WPOI), lymphocytic host response (LHR) and perineural invasion (PNI). All 3 parameters are scored and the total points from the 3 variables are added. If the total score = 0 this is considered to be low risk, if the score is 1 or 2, this is considered intermediate risk and if the score is > 3, this is categorised as high risk. The risk model has been shown to correlate significantly with locoregional recurrence (*p* = 0.0004) and overall survival (*p* = 0.0001); particularly in low stage (T1, T2) oral carcinomas. It is suggested that high risk low stage oral carcinomas may benefit from adjuvant radiotherapy; even in the case of satisfactory margins [1].

While the risk model was been shown to be predictive of OS and LRR [1]. An association with presence or absence of lymph node metastasis at presentation has not been fully explored. Oral squamous cell carcinoma is primarily treated with surgery which may include cervical lymph node dissection as a standard of care for high stage disease. However, the role of elective neck dissection in low-stage disease with clinically negative nodes is controversial [2, 3].

A recent UK-wide randomized trial indicated that oral cancer patients who have upfront elective neck dissection had improved overall survival, disease specific survival and reduced loco-regional recurrence [4]. Advocates of sentinel node biopsy as a preferable alternative to neck dissection in N0 disease, however, state that up to 70% of elective neck dissections in clinically and radiological N0 early stage disease are negative and serve only to confirm the preoperative staging data with concomitant morbidity risk [5]. Indeed the 2019 study reported more nerve injury associated with neck dissection; however quality of life was largely unaffected.

In patients with lymph node metastasis, the presence of extracapsular spread is the most important adverse prognosticator in OSCC.

Extracapsular spread (ECS) is defined as extension of metastatic tumour beyond the lymph node capsule [6] and is significantly associated with unfavourable histological features such as vascular and perineural invasion, non-cohesive pattern of invasion and close/involved resection margins [3]. A study by Woolgar et al. of 173 positive neck dissections revealed extracapsular spread (ECS) was the greatest predictor of poor prognosis in the stepwise regression model of Cox, especially in cases with involved margins [7]. The Kaplan–Meier survival curves showed patients following surgery with macroscopic ECS were more likely to die within the first year, while patients tended to die during the second year with microscopic ECS. The survival probability by 3 years was similar: 33% and 36% respectively. In a previous study we found an association with high risk score and PNI so could the risk score be used in turn to predict the possibility of ECS [8]. In comparison to older staging systems, the presence of extracapsular spread in a neck dissection for oral squamous cell carcinoma significantly upstages the N stage in the TNM8 [9] such is its prognostic influence.

Our recent series of 134 patients with early stage oral cancer, our group showed significant association between risk score, overall survival and distant metastasis. Furthermore we were able to demonstrate that tumours upstaged to T3 on TNM8 criteria were more likely to demonstrate high risk score [8]. Within the study population, 85/134 patients underwent a neck dissection along with excision of the primary tumour. The primary aim of this study was to determine if applying the histological risk model to the primary tumour excision could be associated with the presence or absence of metastasis in the neck dissection. We also wanted to determine if the risk model has an association with extracapsular spread; the most important prognostic indicator in oral squamous cell carcinoma. Furthermore, could application of the risk model be used to influence decision whether to undertake neck dissection in real life practice?

## Materials and Methods

Eighty-five patients treated for primary OSCC with primary excision including neck dissection diagnosed between 2009 and 2014 were included in this study. All pathology slides were retrieved. The inclusion criteria included complete demographic and clinical data, T1 and T2 (7th edition TNM) OSCC treated with surgery with or without postoperative radiotherapy, availability of paraffin-embedded blocks and follow-up data of at least 5 years for survivors. The institutional review board for human subject research reviewed and approved the study.

Our exclusion criteria comprised of T3 and T4 OSCC, recurrences, secondary tumours, oropharyngeal tumours posterior to Waldeyer’s ring (including tongue base and tonsil), OSCC with a depth of invasion under 1 mm, spindle cell variant of OSCC, adenosquamous carcinoma and other rare variants of OSCC, squamous carcinomas with prominent intraductal component and patients who are seropositive for HIV.

All haemotoxylin and eosin-stained tumour resection specimens were independently reviewed by three pathologists who were blinded to the demographic data and outcomes. The slides were scored according to the three components of the validated risk model score: worst pattern of invasion (WPOI), lymphocytic host response (LHR) and perineural invasion (PNI), and then categorised according to risk level. Individual analysis by Consultant Pathologists was validated by a consensus meeting at a multiheaded microscope. Ethics approval and access to histology slides and clinical data was approved from the tissue governance department (study number SR679). The cases were selected consecutively with unknown outcomes.

Cross tabulations and Chi squared tests were used to assess correlations between histopathological risk model. Categorical data by using frequency counts and percentages. All calculations were done using SPSS.

## Results

The study group comprised of 85 patients with primary T1 (n = 34, 40%) and T2 (n = 51, 60%) OSCC. This consisted of 54 male and 31 female patients, aged between 28 and 88 years (mean age 63.4 SD 13.3) as shown in Table 1.

**Table 1:** Demographic data and T stage of OSCC patients

Number of patients	85
Male	54 (64%)
Female	31 (36%)
Age range	28–88
Mean age (SD)	63.4 (13.3)
Oral cavity	
T1	34 (40%)
T2	51 (60%)

A chi squared test showed that cases with positive lymph nodes showed statistically significant association with the overall risk model score (*X*^2^ = 22.62 *p* = 0.0001) and its individual components WPOI (*X*^2^ = 15.25 *p* = 0.05), LHR (*X*^2^ = 10.44 *p* = 0.03) and PNI (*X*^2^ = 17.02 *p* = 0.0001) in Table 2. Traditional dataset items showing an association with positive nodes included poorly differentiated carcinoma (*X*^2^ = 16.63 *p* = 0.002), perineural invasion (*X*^2^ = 23.07 *p* = 0.0001), lymphovascular invasion (*X*^2^ = 153.35 *p* = 0.001), mixed pattern of invasion (*X*^2^ = 9.93 *p* = 0.004) and depth of invasion (*X*^2^ = 13.67 *p* = 0.0001). In terms of extracapsular spread, only PNI in the risk model showed a significant association (*X*^2^ = 24.96 *p* = 0.0001 respectively) while traditional dataset items including poorly differentiated carcinoma (*X*^2^ = 139.7 *p* = 0.0001), perineural invasion (*X*^2^ = 13.67 *p* = 0.0001, lymphovascular invasion (*X*^2^ = 10.46 *p* = 0.001) were significantly associated (Table 3).

**Table 2:** The overall score categories of the risk model, depth of invasion, differentiation, perineural invasion, vascular invasion and pattern of invasion amongst neck dissection, metastasis, positive ECS and negative ECS

	Cases with neck dissection	Cases with metastasis	Cases with positive nodes with ECS	Cases with negative nodes
	N = 85	N = 43	N = 17	N = 42
Low	4 (5%)	0 (0%)	0 (0%)	4 (100%)
Intermediate	31 (36%)	12 (38%)	4 (33%)	19 (62%)
High	50 (59%)	31 (59%)	13 (76%)	19 (61%)
DOI < 5 mm	12 (14%)	4 (33%)	0	8 (20%)
DOI 5–10 mm	42 (49%)	17 (40%)	8 (47%)	25 (59%)
DOI > 10 mm	31 (36%)	22 (71%)	9 (53%)	9 (21%)
Well differentiated	2 (2%)	0	0	2 (5%)*
Moderately Differentiated	46 (54%)	21 (49%)	7 (41%)	25 (60%)*
Poorly differentiated	37 (44%)	22 (51%)	10 (59%)	15 (36%)*
	P = 0.0001 150.62	P = 0.002 16.630	P = 0.0001 139.701	
PNI+	35 (41%)	22 (51%)	12 (71%)	13 (31%)
PNI−	50 (59%)	21 (49%)	5 (29%)	29 (69%)
	P = 0.0001 155.08	P = 0.0001 23.07	P = 0.0001 153.35	
LVI+	24 (28%)	18 (42%)	9 (53%)	6 (14%)
LVI−	61 (72%)	25 (58%)	8 (47%)	36 (86%)
	P = 0.32 4.57	P = 0.001 13.67	P = 0.001 10.46	
Cohesive	16 (19%)	5 (12%)	1 (6%)	11 (26%)
Non-cohesive	48 (56%)	25 (58%)	10 (59%)	23 (55%)
Mixed	21 (25%)	13 (30%)	6 (35%)	8 (19%)
	P = 0.03 6.89	P = 0.04 9.93		

* percentages do not add up to 100% due to rounding of numbers

**Table 3:** The overall score categories of the risk model metastatic cases with positive ECS

ECS					
Positive ECS n = 17			Negative ECS n = 26		
Low	Intermediate	High	Low	Intermediate	High
0	4 (24%)	13 (76%)	0	8 (30%)	18 (69%)

**Fig. 1:**
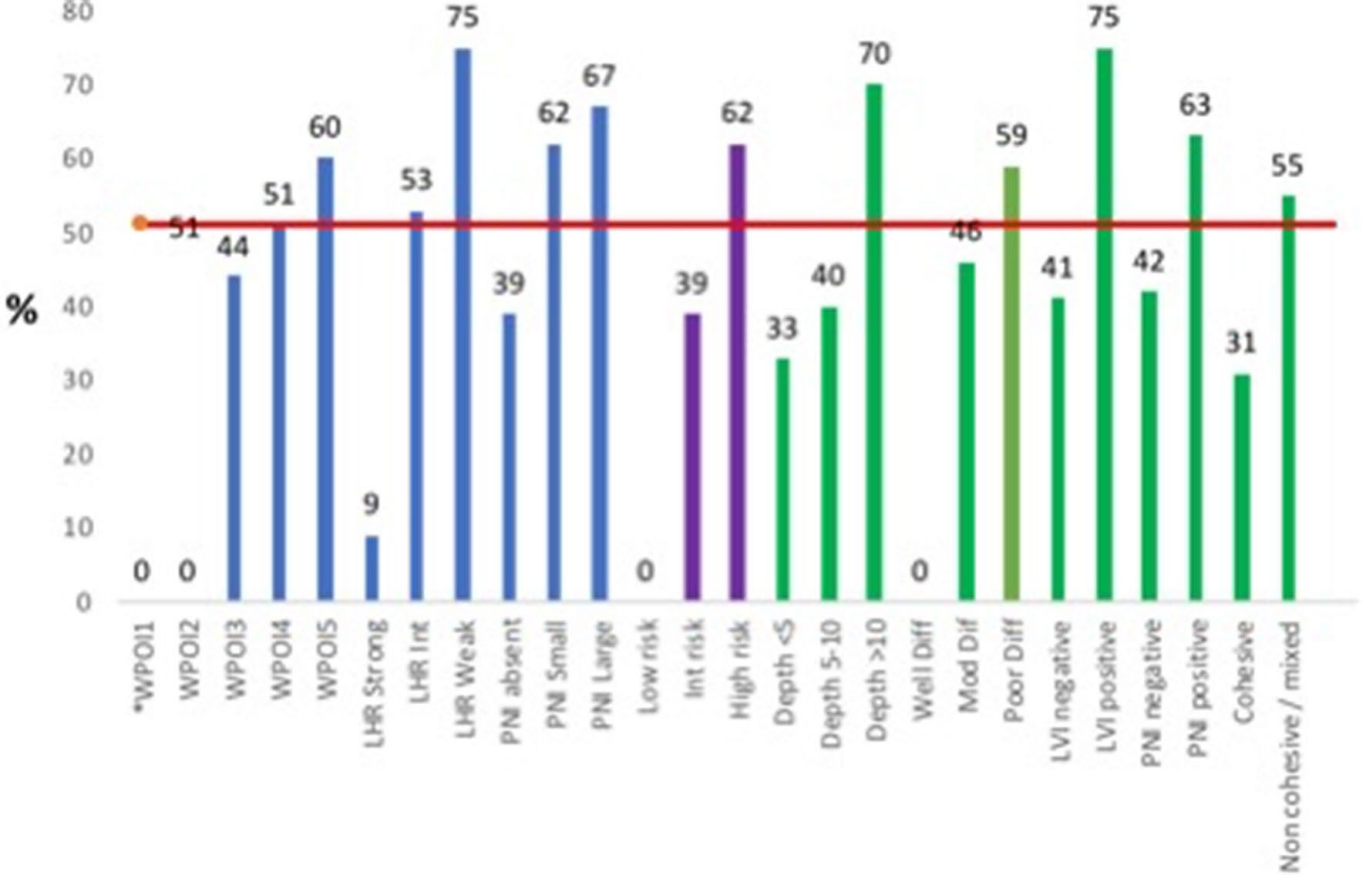
This chart demonstrates the percentage of individual primary tumour characteristics associated with lymph node metastasis. The red horizontal line demonstrates the base rate of metastasis in this series (51%). The areas in blue represent the individual risk model parameters, the areas in purple represent the combined risk scores and the green areas represent the traditional dataset components. In the risk model, tumours with parameters WPOI5, LHR Int, LHR weak, PNI small and PNI large all exhibited a metastasis rate greater than the base rate. In the combined risk score only high risk tumours exhibited a metastasis rate greater than the base rate. In the traditional dataset values poorly differentiated, LVI positive, non-cohesive and PNI positive tumours exhibited a metastasis rate greater than the base rate. Of note tumours demonstrating WPOI2, Low risk and well differentiated tumours exhibited a 0% metastasis rate. *None of the tumours in this series exhibited WPOI1

TNM 7th edition was highly statistically associated with positive nodes and ECS (*X*^2^ = 20.03 *p* = 0.0001, *X*^2^ = 51.58 *p* = 0.0001 respectively). TNM 8th edition was highly statistically positive nodes and ECS (*X*^2^ = 49.66 *p* = 0.0001, *X*^2^ = 15.22 *p* = 0.0001 respectively). Overall risk score was highly statistically significantly associated with TNM 7th edition and TNM 8th edition staging (*X*^2^ = 23.46 *p* = 0.0001, *X*^2^ = 22.94 *p* = 0.001 respectively) (Fig. 1).

The risk score is highly sensitive with low specificity (Table 4). The positive predictive value is low but the negative predictive value is 100% suggesting that the risk score of zero can predict an absence of metastasis.

**Table 4:** The specificity and sensitivity with disease progression

	Disease present	Disease not present	
High (positive)	a 31	b 19	a + b 50
Low (negative)	c 0	d 4	c + d 4
	a + c 31	b + d 23	N 54
High + int (positive)	a 43	b 38	a + b 81
Low (negative)	c 0	d 4	c + d 4
	a + c 43	b + d 42	N 85

## Discussion

The prognostic value of the presence of lymph node metastasis in the management of oral squamous cell carcinoma is universally accepted. The presence of extracapsular spread is of greatest importance with 3-year survival of at least 33% in a large series. 0.5–25% of OSCC patients (with ECS) show evidence of distant metastasis within 2 years of being diagnosed [10]. To put this into context, the 3-year survival of patients without nodal metastasis has been reported as 81%, dropping to 72% with nodal metastasis without extracapsular spread. The presence of extracapsular spread accordingly has been shown to be significantly associated with unfavourable histological features such as vascular invasion, perineural invasion, non-cohesive pattern of invasion and close/involved resection margins [3].

One of the aims of the study was to determine if quantitative risk assessment of the histological features as applied in the risk model could be associated with the presence of neck metastasis and extracapsular spread at the time of presentation. In our study 72% (n = 31) of patients with histologically confirmed nodal metastasis had a combined risk score ≥ 3 (high risk), 28% had risk scores of 1–2 (intermediate risk) and, interestingly, none of cases with a risk score of 0 (low risk, n = 4) showed evidence of metastasis in the neck dissection. Among the cases with positive nodes, 76% of cases with histologically confirmed ECS were shown to be high risk (n = 13) while the remaining ECS cases were intermediate risk (n = 4).

Adding to the sizable body of evidence linking histological risk assessment with clinical outcomes [1, 8, 11–14], we were able to demonstrate statistically significant associations with overall risk score its individual parameters (WPOI, PNI & LHR) with the presence of nodal metastasis in accompanying neck dissection specimens. PNI was significantly associated with the presence of ECS however there was no such association with overall risk score, WPOI or LHR.

Traditional dataset items including depth of invasion, perineural spread, differentiation (poorly differentiated) and lymphovascular invasion were also significantly associated with presence of metastasis in the neck dissection. The results indicate that the risk score could be incorporated into existing reporting practices to provide additional important prognostic information to aid treatment planning, inform patients on prognosis and could potentially help surgeons decide on treatment options. An advantage of the risk model over the traditional dataset values is that it integrates multiple complex histological features into an easily communicable score which is potentially more user friendly for clinicians than transcribing existing extensive parameters from the pathology report. We believe risk score is a useful adjunct however we emphasise that decisions regarding management of the neck should be based primarily on a combination of traditional dataset values and the most recent national/international guidelines.

Indubitably, any idea that risk score could be applied to aid surgical planning of the neck is limited in that application of the risk score can only be valid if the whole tumour interface can be examined histologically (i.e. an excision). This vital principle rests on the fact that risk scoring an incisional biopsy may be potentially misleading due to enormous variation in LHR and POI over a whole tumour volume [13] (although a finding of PNI and/or WPOI5 in an incisional biopsy would, logically, almost certainly have a high-risk score in subsequent excision).

As neck dissections often accompany primary excisions then this would render application of risk score to determine status of the neck as essentially redundant. Potential exists however with the advent of sentinel node biopsy in oral squamous cell carcinoma. Recent meta analyses have shown sentinel node biopsy to have encouragingly high sensitivity (88% [15] and 83% [16]) and specificity (99% [15] and 98% [16]) with greater sensitivity and specificity in the studies employing immunohistochemistry [16]. Avoidance of false positives is paramount to success with this technique and, even with such promising sensitivity and specificity, it is of note that a major Europe-wide trial reported a false negative rate of 14% [17]. Given the close association of tumours with high risk score and nodal metastasis, establishing the risk score of sentinel-node-negative primary oral cancers may justify consideration for elective neck dissection in the case of tumours with high risk score—particularly in cases where further surgery may needed (primary tumours with unexpectedly close margins due to aggressive pathological features for example). More studies are needed in this area.

As stated above, none of the tumours with a risk score of zero showed evidence of metastasis in the accompanying neck dissection (NPV = 100%) perhaps indicating the metastatic potential of these tumours is low. This correlates with our previous observation that low risk tumours showed excellent overall survival with none of the low patients in our original series dying of disease or showing evidence of disease progression after treatment [8]. These findings should be interpreted with a degree caution as low risk cases made up just 4.7% of the cases in the current series (n = 4) and 6% (n = 8/134) of the original series. It should be noted, however, that low risk tumours are rare entities constituting 6–13% of other studies [1, 8, 18]. Therefore to truly test this hypothesis would require analysis of an exceedingly large series. Any proposition that low-stage oral squamous cell carcinoma with a risk score of zero may represent a rare variant with excellent long-term survival and low metastatic potential needs further study.

A limitation of our study is that it was not possible to retrieve the clinical N stage for all our patients at presentation. Ideally, a study investigating risk score in patients with clinically N0 necks to correlate with presence or absence of metastasis would have been of greatest value. However, the aims of this study were to explore potential histological trends between primary tumours that show metastasis versus those that do not.

## Conclusion

Risk assessment of low-stage oral squamous cell carcinoma primary tumours may be predictive of the presence or absence of metastasis at presentation. Knowledge of the risk score and its constituent parts may inform treatment decisions at multidisciplinary meetings. Low risk squamous cell carcinoma may be a rare variant with low metastatic potential and excellent long-term survival.

## Abbreviations

OSCC: Oral squamous cell carcinoma
POI: Pattern of invasion
WPOI: Worst pattern of invasion
TIL: Tumour infiltrate lymphocyte
LHR: Lymphocytic host response
PNI: Perineural invasion
ECS: Extracapsular spread
